# Can probiotics mitigate age-related neuroinflammation leading to improved cognitive outcomes?

**DOI:** 10.3389/fnut.2022.1012076

**Published:** 2022-11-24

**Authors:** R. C. Anderson

**Affiliations:** Te Ohu Rangahau Kai, AgResearch, Palmerston North, New Zealand

**Keywords:** neurodegeneration, mild cognitive impairment, Alzheimer's disease, dementia, gut permeability, microbiota, probiotics, neuroinflammation

## Abstract

Changes in brain structure and cognitive function are a natural part of aging; however, in some cases these changes are more severe resulting in mild cognitive impairment (MCI) or Alzheimer's disease (AD). Evidence is mounting to show that neuroinflammation is an underlying risk factor for neurodegenerative disease progression. Age-related neuroinflammation does not appear to occur in isolation and is part of increased systemic inflammation, which may in turn be triggered by changes in the gut associated with aging. These include an increase in gut permeability, which allows immune triggering compounds into the body, and alterations in gut microbiota composition leading to dysbiosis. It therefore follows that, treatments that can maintain healthy gut function may reduce inflammation and protect against, or improve, symptoms of age-associated neurodegeneration. The aim of this mini review was to evaluate whether probiotics could be used for this purpose. The analysis concluded that there is preliminary evidence to suggest that specific probiotics may improve cognitive function, particularly in those with MCI; however, this is not yet convincing and larger, multilocation, studies focus on the effects of probiotics alone are required. In addition, studies that combine assessment of cognition alongside analysis of inflammatory biomarkers and gut function are needed. Immense gains could be made to the quality of life of the aging population should the hypothesis be proven to be correct.

## Introduction

As part of the natural process of aging, changes in brain pathology occur, which lead to a gradual decline in cognitive function. In some cases, this process is accelerated, leading to a condition known as mild cognitive impairment (MCI). In people with MCI, cognitive ability is decreased to a greater extent than in normal aging, but the ability to function in everyday life is not lost (1). In contrast, Alzheimer's disease (AD) is a neurodegenerative disorder characterized by an on-going decline in cognition that limits the person's ability to function independently. Although MCI is a risk factor for AD, this development is not inevitable, and in fact recent evidence indicates that MCI can be reversed (2).

The pathogenesis of MCI and AD are multifaceted, but one of drivers thought to influence the progression of normal aging to MCI, and MCI to AD, is the presence of neuroinflammation (3), which is chronic inflammation of the central nervous system. Aging is also associated with chronic mild systemic inflammation (4), and changes in gut function, including alterations in gut microbiota composition and increased gut permeability (5). It has been suggested that these age-related physiological changes are interconnected, and that gut dysfunction may be a driver of systemic inflammation, and in turn neuroinflammation (6, 7). If this is indeed the case, then treatments that can maintain proper gut function, may reduce neuroinflammation and protect against, or improve symptoms of, age-associated neurodegeneration. One potential treatment is probiotics, which are beneficial bacteria, that in some cases can reduce intestinal permeability and have anti-inflammatory effects (8, 9).

This mini review briefly summarizes current knowledge on the role of neuroinflammation in age-related cognitive decline and how age-related changes in gut function may lead to neuroinflammation. Then, the mini review focuses on clinical studies assessing the ability of probiotics to improve measures of cognition during aging or as a treatment for MCI or AD.

## The role of neuroinflammation in age-related cognitive decline

Neuroinflammation is an inflammatory response in the brain or spinal cord. Although an inflammatory response is vital to fight infections, the term neuroinflammation is generally used to describe an unnecessary and detrimental immune response (3). Chronic neuroinflammation is thought to be a key factor in the progression of neurodegenerative diseases (10).

During the normal aging process in healthy adults, changes in cognition and brain structure occur, which are more pronounced in those with MCI. Cognitive changes include the progressive decrease in reasoning, spatial visualization, memory and speed, alongside retained, or possibly improved, vocabulary knowledge (11). People with MCI have isolated cognitive impairment, commonly in verbal episodic memory, that does not interfere with their ability to carry out everyday tasks (12). Changes in brain structure with aging include reduced brain volume and increase in ventricular size, as well as cortical thinning and white matter volume reduction that is most apparent in the frontal lobe (13). A meta-analysis showed that across studies brain atrophy was 0.46% per year higher in those with MCI compared to normal aging (14). In addition, the volume loss in key memory and cognition regions, the hippocampus and enroehinal cortex, were both 1.35% per year higher in people with MCI.

The underlying cellular and molecular causes of the age-associated changes in brain structure and the resulting loss of cognition are multifaceted. Mattson and Arumugam (15) describe the nine hallmarks of an aging brain, one of which is neuroinflammation. As discussed by Norden and Godbout (16), aged-related neuroinflammation is characterized by an increase in levels of proinflammatory cytokines, such as interleukin (IL)-1β, and a decrease in levels of anti-inflammatory cytokines, such as IL-10 and IL-4. This inflammatory profile appears to be the result of both glial cells and astrocytes being activated in the aged brain. This activation coincides with increased systematic inflammation which may trigger the neuroinflammation in turn impair cognition (17). In support of this, a recent systematic review indicates that there is an association between an increase in peripheral inflammation markers and poorer cognition in aging (18). However, further research is required to understand prove the cause and effect.

In the case of AD, which is a progressive degenerative brain disorder, where cognition is reduced to the extent that it interferes normal daily tasks, the molecular characteristics are well-studied. These are the build-up of β-amyloid plagues formed by the aggregation of β-amyloid peptides outside the neurons and the build-up of neurofibrillary tangles formed by hyperphosphorylated tau proteins inside the neurons (19). β-amyloid plaques interfere with neuronal communication at synapses which triggers cell death, whereas tau tangles block the transport of molecules inside neurons. These two abnormalities interact in such a way that once a critical amount of β-amyloid plaques are formed, abnormal tau tangles spread throughout the brain, causing the disease progression (19). This eventually leads to excessive brain atrophy and severe dementia.

Although the appearance of β-amyloid plaques and tau tangles are often not apparent until neurodegeneration is observed, the aberrant processes, including excessive neuroinflammation, are thought to start 20 years before symptom onset (20). Initially, the toxic β-amyloid plaques and tau tangles proteins can be cleared by the microglial cells in the brain. However, this causes the microglia to be activated and release pro-inflammatory cytokines. This then makes it more difficult for the microglia to keep up with removing the unwanted proteins, leading to excessive neuroinflammation which when it reaches a threshold initiates glial cells to destroy neurons. The ability for glial cells to adequately keep up with the removal of β-amyloid plagues and tau tangles is likely related to the underlying neuroinflammation present which determines the leeway before the threshold is reached. It is proposed that this baseline neuroinflammation level is influenced by the systemic inflammation in the body (21).

## Associations between age-related changes in the gut and neuroinflammation

As described above, systemic inflammation, which leads to neuroinflammation, is likely a risk factor for the progression of neurodegenerative diseases. Although systemic inflammation can be the result of numerous processes and diseases, one key trigger during aging is changes in the gut, such as the increase in gut permeability and alterations in microbiota composition (22).

During aging, the gut naturally becomes more permeable (23). The gastrointestinal tract, with a surface area of approximately 32 m^2^ in an adult human, is the second largest interface between the host and the outside world (24). Therefore, this reduction in its barrier function associated with aging has follow-on consequences, such as allowing unwanted components (antigens and opportunistic pathogenic bacteria) to enter the body, leading to the production of proinflammatory cytokines in the blood. These cytokines can then cross the blood brain barrier to cause neuroinflammation, which impacts brain function (25).

Whether age-associated increases in gut permeability are a cause or a consequence of other gut related changes is currently unknown. However, a recent study showed that the development of gut barrier dysfunction during aging is not consistent across all people (26). People with irritable bowel syndrome (IBS) are more susceptible to gut hyperpermeability in later life than healthy individuals (26). Interestingly, those with IBS are also more at risk at developing AD (27), supporting the idea that gut hyperpermeability is a risk factor for AD.

Current data on the association of gut barrier dysfunction with age-related neurodegeneration is conflicting. In one study, the blood biomarker zonulin, which is a group of structurally and functionally related proteins that are required for maintaining intestinal barrier integrity, was quantified. Zonulin was higher in people with MCI compared to healthy controls, and higher again in people with AD compared to those with MCI (28). Zonulin concentration was also associated with lower scores in the Mini-Mental State Examination (MMSE). In contrast, another study did not detect any difference in gut permeability between healthy controls, and participants with MCI and AD (29). However, as the authors acknowledge, the blood biomarkers used were lipopolysaccharide binding protein (LBP) and intestinal fatty acid binding protein (IFABP), which are measures of acute inflammation and therefore more indirect measures of intestinal permeability than zonulin.

Another aspect of the gut that changes with aging is the gut microbiota composition. The human gut is host to approximately 10^13^ bacterial cells which are collectively referred to as the gut microbiota (30). Over 2000 species of bacteria have been isolated from human gut samples, with the majority of these belonging to four main phyla (31). The composition of gut microbiota varies among individuals, based factors such as diet, lifestyle, geographical location, and health status, as well as age.

Recent studies have sought to define a microbiota profile associated with healthy aging, but this varies between the study populations (32, 33). However, as reviewed by Wu et al. (34), gut microbiota composition of people with AD has been shown to differ from that of healthy older adults in a number of studies. Recent studies have also shown there is a difference in the composition of the gut microbiota between people with MCI and those without (35), and that these differences are similar to those seen in people with AD (36). This implies that microbiota dysbiosis proceeds AD development and, therefore, may be a driver in the disease progression.

A key consequence of microbiota dysbiosis is changes in microbial metabolite production, and in particular a reduction in beneficials short-chain fatty-acids (SCFAs). For example, some SCFAs are known to support intestinal barrier function, and these SCFAs, as well as the microbes that produce them, have been found to be in higher abundance in healthy older adults vs. those who are frail or experiencing cognitive impairment (37). In addition, in older adults with cognitive performance from average to AD, anti-inflammatory SCFA concentrations were positively correlated with cognitive performance; whereas as pro-inflammatory microbial lipopolysaccharide concentrations were associated with poorer cognition (38). SCFAs downregulate the expression of pro-inflammatory cytokines, such as IL-1β which is associated with not only increased intestinal barrier permeability (39), but also blood brain barrier permeability (40) and a glial cell inflammatory state (41). Therefore, changes in microbiota composition that result in a decrease in SCFA production impact inflammation systemically.

Microbial-derived SCFAs impact host inflammatory pathways *via* epigenetic mechanisms because they are inhibitors of histone deacetylases (HDACs) (42). HDACs repress transcription by removing acetyl-groups from histones in DNA; therefore, conversely SCFA enable transcription of these genes. Rodent studies indicate that HDAC activity increases with age and in models of AD, and that this is associated with decreases synaptic plasticity (43); therefore, HDAC inhibitors, such as SCFAs, may have a protective effect. Indeed, the SCFA acetate has been shown to reduce histone acetylation and decrease IL-1β expression in a rodent model of neuroinflammation (44). Together this shows that the products of the gut microbiota can affect neuroinflammation *via* epigenetic mechanisms independent of their effects on gut barrier function.

## The use of probiotics to mitigate age-related cognitive decline

Probiotics are defined as “live microorganisms that, when administered in adequate amounts, confer a health benefit on the host” (45). Probiotics are thought to be able to improve gut barrier function, alter microbiota composition and reduce systemic inflammation, all of which may lead to a reduction in neuroinflammation. The concept of using probiotics in the prevention or treatment of age-related neurodegenerative diseases is discussed widely in the literature in review articles and opinion pieces, however, to date there are few original research articles to support this. A Scopus search of [(“ageing” OR “mild cognitive impairment” OR “Alzheimer's disease”) AND “probiotics”] resulted in 420 articles which was limited to 187 based on screening the titles to exclude articles that were off topic. These abstracts were reviewed to select primary research on probiotic intervention with older adults. Review articles and research articles using animal or cell models were excluded. Nine human clinical studies for which there is sufficient information to critique the findings are discussed below. Studies with quasi-experimental designs were excluded from this review.

### Probiotic treatment for healthy older adults

There are two published studies that investigated the effects of mixes of bifidobacteria on cognition in healthy older adults. Although the first study had some limitations, the second provided preliminary evidence that probiotics may improve cognitive function in older adults *via* modulation of the gut microbiota.

In the first study, all participants (66–78 years) underwent a 12-week resistance training programme. One group (*n* = 20) also received a multi-strain probiotic mixture (*Bifidobacterium longum* subsp. *longum* BB536, *B. longum* subsp. *infantis* M-63, *Bifidobacterium breve* M-16V and *B. breve* B-3) and the other group did not (*n* = 18) (46). Both groups had an increase in cognitive function, as measured by the Japanese version of the Montreal Cognitive Assessment (MoCA-J), but there was no difference in the improvement of those receiving the probiotics verses those who did not. There was potentially a larger improvement in the number of correct responses in the Flanker test in the probiotic group, however, this was not statistically significant (*P* = 0.056). This study focused on the combination of exercise and probiotics, therefore there was not a treatment group where participants received the probiotic without the resistance training, and no control group that did not receive either the resistance training or probiotic. The study did not assess any gut-related parameters, however, this probiotic combination has previously been shown to be effective in children with IBS (47), so it is plausible that it is having its cognitive effects *via* alterations in gut function.

In the second study, participants (≥65 years) took placebo (*n* = 31) or probiotic (*n* = 32) (*Bifidobacterium bifidum* BGN4 and *B. longum* BORI) capsules twice a day for 12 weeks (48). *B. bifidum* BGN4 has anti-inflammatory properties and has been shown to be beneficial for infants with eczema and adults with (IBS) (49). In the study by Kim et al. (48), participants in the probiotic group had an increase in mental flexibility (attention and executive function) after 12 weeks compared to baseline, and at 12 weeks compared to the placebo group, as measured by the Korean version of the Consortium to Establish a Registry for Alzheimer's Disease (CERAD-K) test. In addition, after 12 weeks the probiotic group had higher blood serum levels of brain-derived neurotrophic factor (BDNF), which is a protein known to protect against neuroinflammation. BDNF levels correlated with increased in abundance of particular gut bacteria subpopulations (*Eubacterium* and Clostridiales), which indicates that the neuroprotective effects may have originated in the gut.

### Probiotic treatment for mild cognitive impairment

There are four published studies that assessed the effects of probiotics in people with age-related MCI and all indicated that the probiotic treatments resulted in improved cognitive function. Two studies used *B. breve* A1 and the other two studies used strains of lactobacilli. There are some indicators that improvements in cognition were related to a reduction in inflammation, however, no gut-related parameters were measured in any of the studies.

Evidence for the effectiveness of the probiotic *B. breve* A1 for improving cognitive function in people with MCI has been accumulated over three publications by the same group. In the first study, participants (≥65 years) with memory complaints were recruited and were given either a probiotic supplement containing *B. breve* A1 (*n* = 32) or a placebo (*n* = 31) for 12 weeks (50). Initial analysis of the data did not show any differences in the improvement in cognitive function compared to baseline scores between treatments groups. However, when the participants were stratified, based on their initial scores in the Japanese version of the Repeatable Battery for the Assessment of Neuropsychological Status (RBANS), those with MCI had greater improvements in MMSE scores after taking the probiotic compared to the placebo.

To build on the previous results, in the second study participants (50–79 years) were selected based on their pre-screening RBANS score being in the range expected for MCI and then assigned to either the probiotic supplement *B. breve* A1 (*n* = 40) or a placebo (*n* = 40) group for 16 weeks (51). After the treatment, the probiotic group had a significant increase in RBANS score compared to baseline and compared to the placebo group, which was driven by improvements in immediate memory, visuospatial/constructional and delayed memory domain scores. The probiotic group also had an increase in MCI screen score. Further analysis of blood biomarkers found that RBANS scores were negatively correlated with hemoglobin A1c (HbA1c) levels (52). This is consistent with the idea that HbA1c levels are associated with the development of neuroinflammation and cognitive decline (53). Interestingly, in the analysis by Bernier et al. (52), participants with higher baseline HbA1c levels had more marked improvement in RBANS scores across more domains of the battery when treated with *B. breve* A1, indicating that the probiotic was able to mitigate some of the negative impacts of HbA1c. In a study using a mouse model of AD, *B. breve* A1 improved cognition and reduced neuroinflammation, however, it did not alter microbiota composition (54). Therefore, further research is needed to understand the mechanisms by which the probiotic present in the gut affects the brain.

In the first study with a lactobacilli, participants (55–85 years) with MCI were treated with a nutritional supplement containing *Lactobacillus plantarum* C29-fermented soybean (*n* = 50) or a placebo (*n* = 50) for 12 weeks (55). *L. plantarum* C29-fermented soybean was previously shown to improve cognition in a mouse model of AD, and that this correlated with changes in brain and blood inflammatory biomarkers and microbiota composition (56). In the study by Hwang et al. (55), the group receiving the nutritional supplement had a greater increase in combined cognitive function after the treatment period than those in the control group, which was largely driven by an increase in attention. For the group receiving the nutritional supplement, there was a positive correlation between BNDF levels and increase in cognitive function, whereas there was no relationship between the two measures for the placebo group, which indicates a potential mechanism of action of the supplement. Unfortunately, due to the study design, it is not possible to discern whether the benefits were due to the probiotic, the soybean or the combination of the two.

In the second study with a lactobacilli, the effects of the well-known probiotic *Lactobacillus rhamnosus* GG on cognition in middle-aged and older adults (52–75 years) was assessed (57). Participants were randomly assigned to consume placebo (*n* = 68) or probiotic (*n* = 77) capsules daily for 3 months, and cognitive ability was assessed before and after the treatment using the NIH Toolbox of Neurological and Behavior Function. Similar to the results of the Kobayashi et al. (50) study, greater cognitive improvement was observed in participants with MCI taking the probiotic than those with MCI taking the placebo, and this treatment effect was not apparent for those with intact cognition. The physiological mechanisms for the change in cognition were not explored in this study. However, *L. rhamnosus* GG is a well-studied probiotic with 30 years of research to illustrate it gut health benefits and anti-inflammatory properties (58), so it is plausible that it acts *via* the gut-brain axis.

### Probiotic treatment for Alzheimer's disease

There are three published studies that investigate the effects of probiotics on cognitive function in people with AD. These studies were all conducted by the same research group and used mixed probiotic cultures containing both bifidobacteria and lactobacilli. All of the studies have limitations, and the results were varying. Like the MCI studies, some indicators of systemic inflammation were measured, but the link to gut function was not explored. Furthermore, the strain names for the probiotics used were not given so it was not possible to determine if they had known gut health benefits.

In the first study, participants (age range not given) received either a probiotic milk drink containing *Lactobacillus acidophilus, Lactobacillus casei, B. bifidum*, and *Lactobacillus fermentum* (*n* = 30) or standard milk (*n* = 30) for 12 weeks (59). The authors reported a 28% increase in MMSE score after the probiotic treatment compared to no increase in the control group. In addition, they reported changes in a range of metabolism related biomarkers, but no changes in biomarkers related to oxidative stress and inflammation. Unfortunately, the validity of this study is currently under review by the institute in which it was conducted, so the results should be viewed with caution until this matter is resolved (60).

In a follow up study, participants (65–90 years) in the probiotic treatment group (*n* = 25) received two different supplements on alternate days for 12 weeks; one containing *L. fermentum, L. plantarum*, and *Bifidobacterium lactis* and one containing *L. acidophilus, B. bifidum, and B. longum* (61). There was also a placebo group (*n* = 23) which received maltodextrin capsules. Unlike the previous study, the probiotic treatment group did not have a significant increase in cognitive function after the treatment. The authors proposed that this was due to the severity of AD in the participants, which was higher than in the previous study. It is also worth noting that cognitive function was assessed using a different measure - the Test Your Memory (TYM) brief test instead of the MMSE - which may also have also accounted for the difference in results.

In the third AD study, Tamtaji et al. (62) compared participants (55–100 years) treated with a placebo (*n* = 26), a selenium supplement (*n* = 26), or a selenium supplement plus a probiotic mixture containing *L. acidophilus, B. bifidum* and *B. longum* (*n* = 27) for 12 weeks. The group receiving the selenium plus probiotic supplement had a significant increase in MMSE score, whereas the placebo and selenium only groups did not. Differences in some inflammatory and oxidative stress-related biomarkers were also observed in the group receiving the probiotic supplement compared to the other groups. These included an increase in blood plasma concentrations of glutathione (GSH), which is important in reducing oxidative stress, as well as total antioxidant capacity (TAC), and a reduction in the lymphocyte gene expression of the pro-inflammatory cytokine tumor necrosis factor alpha (TNFβ). As the focus of this study was on probiotic and selenium co-supplementation, there was not a group which received the probiotic mixture only.

## Conclusions

The concept that neuroinflammation is a critical driver of neurogenerative disease progression is discussed widely in the scientific literature and the idea that systematic inflammation is a risk factor for this is gaining traction. However, cognitive impairment during aging is a complicated and multifaceted process, and the contribution of various factors and their interplay is yet to be fully understood. There is another layer of complexity when considering the hypothesis that systemic inflammation that triggers neuroinflammation is initiated in the gut and that this process may be mitigated using probiotics. There is much discussion and conjecture in the literature, but very little original research.

The answer to the question this minireview sought to investigate—*Can probiotics mitigate age-related neuroinflammation leading to improved cognitive outcomes?*—remains unknown. There is preliminary evidence to suggest that specific probiotics may improve cognitive function, particularly in those with MCI; however, the results are not just conclusive. Larger, multilocation studies designed specific to assess the effect of probiotics on cognition alongside the analysis of biomarkers of systemic inflammation, gut permeability and microbiota composition are needed.

## Author contributions

RA completed the literature search and wrote the mini review.

## Funding

This mini review was partially funded by the New Zealand Ministry of Business, Innovation & Employment as part of the Endeavor Research Programme entitled Smarter Lives: New Opportunities for Dairy Products Across the Lifespan (Contract C10X1706).

## Conflict of interest

The author declares that the research was conducted in the absence of any commercial or financial relationships that could be construed as a potential conflict of interest.

## Publisher's note

All claims expressed in this article are solely those of the authors and do not necessarily represent those of their affiliated organizations, or those of the publisher, the editors and the reviewers. Any product that may be evaluated in this article, or claim that may be made by its manufacturer, is not guaranteed or endorsed by the publisher.
